# Extensive Aortic Dissection in a Low-Risk Male Causing Acute Kidney Injury: A Case Report

**DOI:** 10.7759/cureus.46283

**Published:** 2023-09-30

**Authors:** Francis C Anene

**Affiliations:** 1 Accident and Emergency, Darlington Memorial Hospital, Darlington, GBR

**Keywords:** acute kidney injury, hypertension, ct angiogram, chest pain, aortic dissection, stanford type a

## Abstract

Aortic dissection is a major differential diagnosis in an elderly male with severe chest pain radiating to the back who has a history of hypertension, smoking, or connective tissue disorders such as Marfan and Ehlers-Danlos syndromes. It is a medical emergency with a high mortality rate if undetected and untreated. This report describes the case of a patient presenting with extensive aortic dissection with no significant risk factors who was diagnosed following a CT angiogram of the aorta. He was subsequently managed medically before being transferred for definitive surgical management with a good outcome.

## Introduction

Aortic dissection describes the condition when a separation has occurred in the aortic wall intima, causing blood flow into a new false channel composed of the inner and outer layers of the media. Dissection most commonly occurs with a discrete intimal tear but can occur without one. An aortic dissection is considered acute if the process is less than 14 days old [1]. In acute aortic dissection (AAD), this compromise in the layer integrity of the vessel wall creates a false lumen between the intima and media layers through which blood leaks proximally and distally, presenting clinically as chest pain. This pain has been classically described as 'tearing', and radiates to the back.

In the younger age group, it is an extremely rare and difficult diagnosis to make, especially in the absence of other telltale features. The severity of presenting signs and symptoms generally depends on the extent of involvement of arterial branches in the organs and their degree of perfusion from true or false lumens [2]. Physicians correctly suspected the diagnosis in only as few as 15%-43% of confirmed cases of AAD. If untreated, mortality is close to 50% in the first 48 hours [3].

The Office for National Statistics (ONS) data for the population of the UK in mid-2020 was 67 million, of which 59,597,300 people resided in England and Wales. There were 4,106 deaths registered as aortic aneurysm and dissection (0.007%), which is equivalent to seven per 100,000 population. The average age was over 60 years, and the majority were male [4].

Aortic dissection is classified into two types: A, involving the ascending aorta proximal to the origin of the brachiocephalic artery, and B, involving only the descending aorta. This is the Stanford classification. A second classification is the DeBakey classification, which consists of three types. Type 1 originates in the ascending aorta and at least the aortic arch; type 2 originates in and is confined to the aortic arch; and type 3 arises from the descending aorta and extends distally proximal and distal to the diaphragm [2]. This report makes reference to the Stanford classification.

## Case presentation

A 50-year-old male presented to the emergency department with severe central chest pain radiating to his left shoulder and neck. He had been awakened by the pain, which he described as sharp initially, then dull, radiating to his back. At the time of assessment, he had been in pain for over 10 hours and had no relief with paracetamol and oral morphine given in triage. He was a non-smoker and had no personal or family history of hypertension or connective tissue disease. Examination revealed a blood pressure of 142/95 mmHg, with no significant difference in both arms and no radial pulse deficit. His oxygen saturation was 97% (room air) with clear lung fields. There were no heart murmurs and no focal neurological deficits. The differential diagnoses at this time were acute coronary syndrome and acute aortic dissection.

The ECG showed a normal sinus rhythm. Blood revealed a titration of 20 ng/L at first and a repeat troponin of 25 ng/L two hours later. Serum creatinine was raised at 113 mmol/L. There was no history of kidney disease in the patient. The D-dimer test was not done.

**Figure 1:**
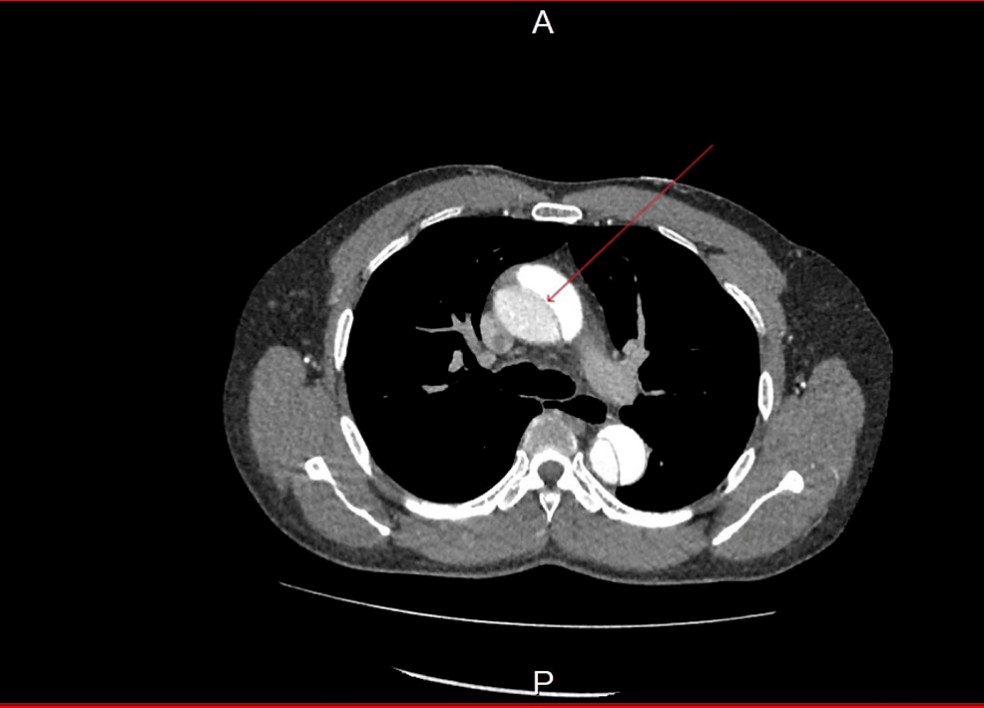
A CT angiogram of the aorta shows dissection of the ascending aorta.

A CT angiogram showed a large Stanford type A aortic dissection extending from the ascending aorta and into the proximal left internal iliac artery. The right renal artery and inferior mesenteric artery (IMA) appeared to come from the false lumen. Coeliac, superior mesenteric artery (SMA), and left renal arteries were coming from the true lumen. Images from his CT angiogram are shown. Figure 1 shows the origin of the dissection from the ascending aorta (Stanford type A).

**Figure 2:**
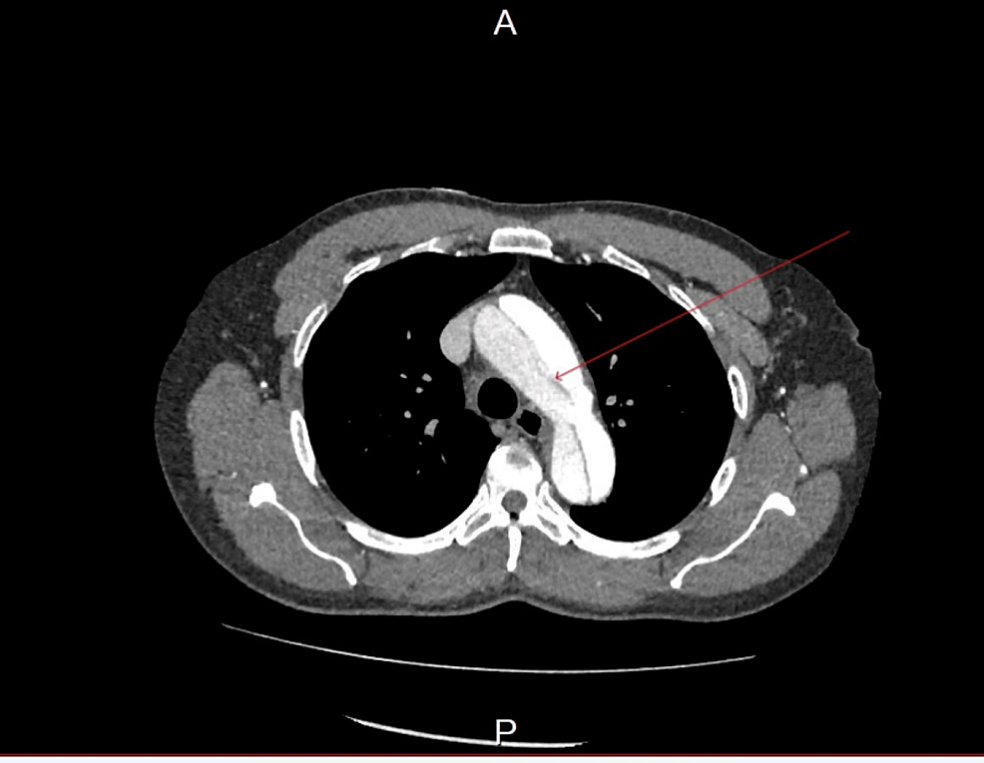
A CT angiogram of the aorta shows dissection of the arch of the aorta.

Figure 2 shows a dissection of the arch of the aorta.

The patient was quickly prepared for transfer to a cardiothoracic center after discussions with the cardiothoracic surgeons. An intravenous labetalol infusion was given to control his blood pressure, with a target systolic blood pressure of 110 mmHg, prior to transfer to a cardiothoracic center. An open surgical repair was done the same day, and the patient made a remarkable recovery to discharge.

## Discussion

**Figure 3:**
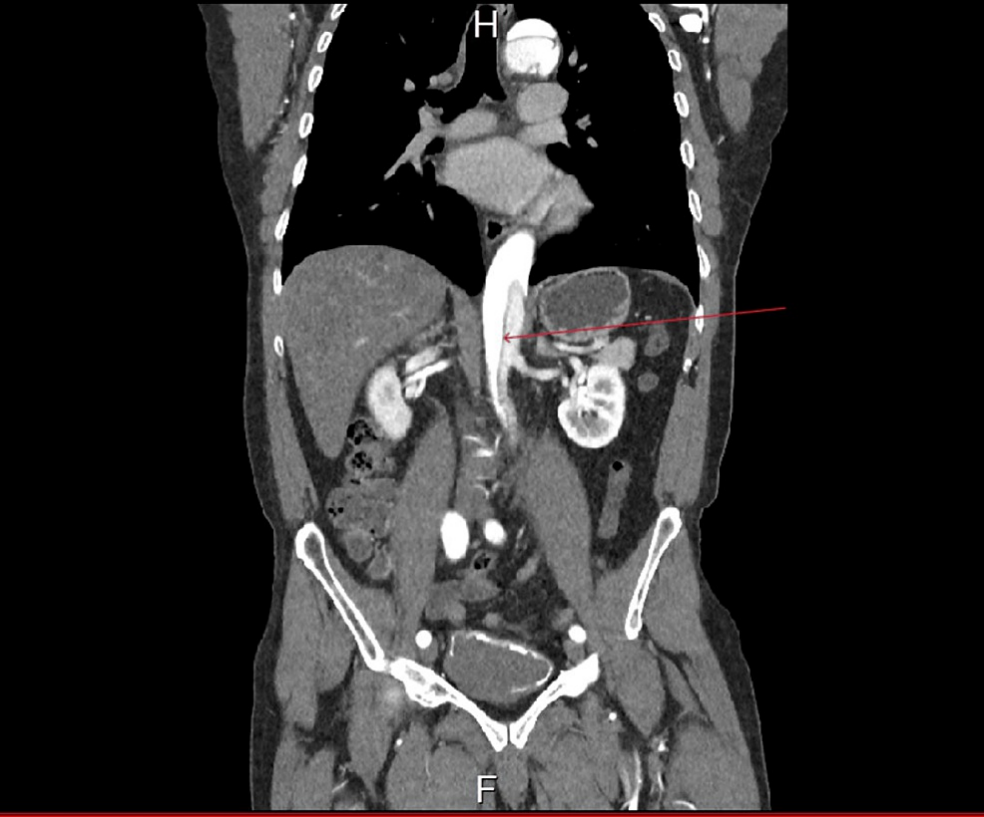
Abdominal aortic dissection in the region of the renal arteries

The risk factors for aortic dissection are well documented and include advanced age >65 years, male sex, hypertension, smoking, and connective tissue diseases like Marfan syndrome [5]. This patient did not have any of the other risk factors that would strongly suggest an aortic dissection in someone presenting with chest pain. Also, though his blood pressure was minimally raised, other high-risk examination findings, like inter-arm variation in blood pressure, cardiac murmurs, and neurological deficits, were absent. With these negatives notwithstanding, a presentation of severe, sudden-onset chest pain radiating to the back should warrant that a diagnosis of acute aortic dissection be seriously considered. More so where the ECG and troponin levels are unremarkable. As this case shows, the absence of well-documented risk factors and high-risk examination findings do not rule out the possibility of extensive aortic dissection. Figure 3 shows the dissection extending into the abdominal aorta, proximal and distal to the origin of the renal arteries.

A CT angiogram of the aorta is the definitive investigation for the diagnosis of an aortic dissection. Where available and clinically expedient, a D-dimer test is the recommended initial investigation of choice for low-risk patients with an aortic dissection detection risk score of 0 or one. It is acknowledged that the patient in this report should have had a D-dimer test done as part of initial investigations. However, having been omitted in triage, it was considered more clinically expeditious to proceed with the more definitive investigation after hours of ongoing chest pain.

**Figure 4:**
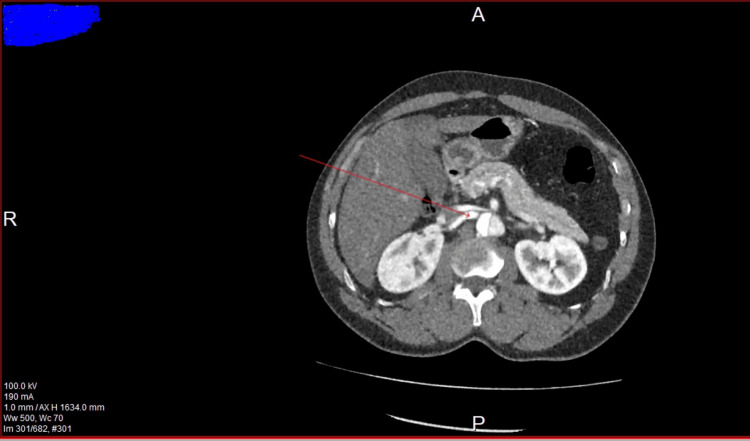
Right renal artery arising from the false aortic lumen

The literature reports a significant correlation between type B aortic dissection and acute kidney injury (AKI) but not much association with Stanford type A aortic dissection. Acute kidney injury is very common in patients with type B aortic dissection, even in clinically uncomplicated diseases [6]. For type A aortic dissections, AKI has been reported as a complication following surgical repair rather than an initial presenting feature [7], making this case all the more unique. The patient's blood results showed a raised creatinine level, which can likely be attributed to renal hypoperfusion as a result of the right renal artery coming off the false lumen and the left coming off the true lumen. Figure 4 shows the right renal artery arising from the false aortic lumen.

The definitive management for acute aortic dissection is emergency vascular surgery. Blood pressure control is an essential therapy for patients with acute aortic dissection and should be maintained throughout the entire treatment. Thus, the vast majority of current guidelines recommend controlling the blood pressure to be lower than 140/90 mmHg. Theoretically, a much lower target may further decrease the risk of the propagation of dissection. However, some argued that very low blood pressure would compromise organ perfusion [8].

## Conclusions

Aortic dissection is a dangerous condition with a high mortality rate when not quickly diagnosed. To diagnose acute aortic dissection, a high index of suspicion is required for patients presenting with high-risk chest pain. Chest pain with back radiation, especially in the absence of both ECG changes and raised troponin levels, should necessitate an investigation for aortic dissection. A CT angiogram is the investigation of choice to conclusively confirm or exclude aortic dissection. As this case shows, the absence of risk factors and high-risk systemic examination findings does not rule out the possibility of significant aortic dissection. A Stanford type A aortic dissection, if missed, will usually result in a high risk of mortality; however, early diagnosis will increase the chances of a successful surgical intervention.
